# Effects of pay for performance in primary care in an under-registration scenario

**DOI:** 10.11606/s1518-8787.2024058005812

**Published:** 2024-10-04

**Authors:** Paula de Castro-Nunes, Paloma Palmieri, Hugo Bellas, Adriana Soares, Jaqueline Viana, Paulo Victor Rodrigues de Carvalho, Alessandro Jatobá

**Affiliations:** I Fundação Oswaldo Cruz Centro de Estudos Estratégicos Antônio Ivo de Carvalho Rio de Janeiro RJ Brasil Fundação Oswaldo Cruz. Centro de Estudos Estratégicos Antônio Ivo de Carvalho. Rio de Janeiro, RJ, Brasil; II Secretaria Municipal de Saúde do Rio de Janeiro Rio de Janeiro RJ Brasil Secretaria Municipal de Saúde do Rio de Janeiro. Rio de Janeiro, RJ, Brasil

**Keywords:** Under-registration, Health Care Financing, Health Systems Financing

## Abstract

**OBJECTIVE:**

To propose a method for detecting and analyzing under-registration and highlight its potential financial effect in view of the implementation of the Previne Brasil Program.

**METHODS:**

An ecological study was carried out to analyze cytopathological exams in programmatic area 3.1 in the municipality of Rio de Janeiro. The data was collected from the *Departamento de Informática do Sistema Único de Saúde* (DATASUS - Department of Informatics of the Unified Health System) database, including information on reports from outsourced cytopathology laboratories and those available in the *Sistema de Informação em Saúde para a Atenção Básica* (SISAB - Health Information System for Primary Care) and the Sistema de Informação do Câncer do Colo do Útero (SISCOLO - Cervical Cancer Information System) of DATASUS/Ministry of Health.

**RESULTS:**

The estimated under-registrations per health unit totaled 108,511 exams in the last two years in the programmatic area 3.1 area, which corresponds to an estimated total of R$ 435,129.00 that would have been foregone if the Previne Brasil Program had been in place during the period studied.

**CONCLUSION:**

The article’s main contribution lies in the presentation of empirical evidence of the potential effects of under-registration on Primary Health Care financing. In addition, there are two other significant findings - firstly, it highlights weaknesses in the process of recording health information inherent to vulnerable regions; secondly, it indicates a vicious circle potentially fueled by sudden changes in Primary Health Care funding conditions, in addition to potential consequences for other levels of care.

## INTRODUCTION

The Previne Brasil Program, established by Ordinance No. 2.979 of November 12, 2019^1^ changed the ways in which resources are passed on to fund Primary Health Care (PHC), which are now distributed based on four criteria: weighted funding; payment for performance; incentives for strategic actions; and financial incentives based on population criteria. In relation to the previous funding model, the transfer based on service performance replaced the fixed primary care floor (PCF), which guaranteed minimum transfers based on the populations covered. This change has not only put pressure on PHC coverage but has also demanded management mechanisms to guarantee the reliability of the recording of procedures carried out by health departments. This is a problem for the most vulnerable territories, in which the recording of health information suffers various disruptions, from difficulties in capturing it to a lack of resources for proper analysis.

Before the Previne Brasil Program was set up, Primary Care funding was made up of the fixed and variable PCF. The value of the fixed PCF was calculated *per capita,* i.e. based on the population estimated by the *Instituto Brasileiro de Geografia e Estatística* (IBGE - Brazilian Institute of Geography and Statistics) in relation to the registered population, ranging from R$ 23.00 to R$ 28.00. The variable PCF, on the other hand, was conditional on the implementation of the Family Health Strategy by municipal managers^2^. Thus, the introduction of Previne Brasil changed the financing of the teams, which meant adopting new procedures for feeding in the data and, consequently, new routines and management processes for PHC professionals^3^.

It is important to note that the records of procedures, exams, consultations, and conduct adopted by health professionals enable communication between members of the multidisciplinary team and the continuity and longitudinality of the care provided to users. By providing direct evidence of health events in the territories, they support the decision-making, planning, execution, and evaluation processes. They are therefore primary parameters for performance pay, even though they are not precise indicators of the expansion of access or the quality of services. In addition, the lack of or incomplete recording of information on the socio-economic profile, the health situation of patients, and the procedures carried out during care is a prevalent and historic problem in the Unified Health System (SUS)^4^.

Under-recording refers to the number of events that should be recorded but end up ignored or lost in health information systems. This can occur in various areas, such as the community, work centers, health services, and the Epidemiological Surveillance System, among others^8^.

The introduction of a performance-based funding model - especially with the discontinuation of the fixed PCF - increases the potential for underfunding, especially in more vulnerable regions or municipalities, which usually face more difficulties in modernizing their management processes, in order to guarantee the reliability of the registry^3^.

In this way, understanding the financial impacts of under-registration on the allocation of PHC resources and informing the agents involved of the need to improve their management is of fundamental importance to enable and guarantee the successful implementation of new pay-for-performance models in the SUS. In this context, the article highlights the potential financial effect of under-registration in the face of the implementation of the Previne Brasil Program, proposing an analysis method in which cytopathological exams in programmatic area (PA) 3.1 of the municipality of Rio de Janeiro are analyzed.

The women’s health line of care, which is directly related to the demand for cytopathology tests, was chosen for this study because it is representative of the importance of qualified information in the articulation between levels of care for the resilience of services. Its correct recording is important not only in terms of possible financial impacts but also to the extent that the capacity of services to respond to problems such as cervical cancer is measured by analyzing the records made at the PHC level. According to data from the National Cancer Institute (INCA)^9,10^ in Brazil, with the exception of non-melanoma skin tumors, cervical cancer is the third most common type of cancer among women. For the year 2023 alone, 17,010 new cases have been estimated.

## METHODS

### Research Design

This is an ecological observational study of a quantitative nature, using data from the management systems of the Municipal Health Department of Rio de Janeiro (SMS-Rio), the data bus of the *Departamento de Informática do Sistema Único de Saúde* (DATASUS - Department of Informatics of the Unified Health System) and outsourced laboratories that provide services to the Ministry of Health of the Research Incentive Fund Association (AFIP) as sources of information. According to the collection procedures, the study was located in PA 3.1 of the municipality of Rio de Janeiro.

This article was prepared in accordance with the recommendations of the EQUATOR Network guidelines, the Strengthening the Reporting of Observational Studies in Epidemiology (STROBE) checklist.^11^.

### Research Environment

The care network in the municipality of Rio de Janeiro is distributed among eight Programmatic Area Coordinators (PACs), covering the entire territory of the municipality (which serves approximately 6,211,423 inhabitants, according to IBGE data): PAC 1.0 (central region); PAC 2.1 (south neighborhoods); PAC 3.1, PAC 3.2, and PAC 3.3 (north neighborhoods); PAC 4.0, PAC 5.2, and PAC 5.3 (west region).

The research was carried out in PAC 3.1, chosen because it is representative of the whole and because it reflects the health and vulnerability context of the municipality of Rio de Janeiro. In addition, this PA has 32 health units and the largest number of teams (218), and has large areas of social, economic, and environmental vulnerability and social inequality, as it encompasses three large favela complexes. PA 3.1 covers an area of 85.36 km^2^, with a population density of 10,386 inhabitants/km^2^, and has 886,551 residents, 80% of whom are covered by the Family Health Strategy, with a total of 714,598 registered.

Among the areas covered by PAC 3.1, there is latent inequality, exemplified by Jardim Guanabara - with a human development index (HDI) of 0.963, in third place among the 126 neighborhoods in the municipality of Rio de Janeiro - and Complexo do Alemão, in 126^th^ place (last) with 0.711. When analyzing the social development index (SDI) - which includes the dimensions of basic sanitation, garbage collection service, toilets for residents, illiteracy, and average income - the region has an index of 0.518, covering the most vulnerable areas of the city.

### Sources of Information

The data used in this study was collected from the PAC 3.1 and SMS-Rio management systems and is also freely accessible through the DATASUS data bus and the outsourced laboratory service provider (AFIP). Information relating to the process of evaluation, control, regulation of care planning and financial management was included, such as the reports from the outsourced laboratories relating to cytopathological tests and those available on the *Sistema de Informação em Saúde para a Atenção Básica* (SISAB - Health Information System for Primary Care) and the *Sistema de Informação do Câncer do Colo do Útero* (SISCOLO - Cervical Cancer Information System) of DATASUS/Ministry of Health, as well as data collected directly from SMS-Rio’s TabNet.

### Data Collection Procedure

Data on the number of cytopathology tests carried out by the 32 PHC units under the management of PAC 3.1 of the SMS-Rio was collected from the information department. The data refers to:

- number of tests requested and recorded for female patients aged 25 to 64 living in the municipality of Rio de Janeiro, organized by health unit, month, and year;
- amounts paid to third-party laboratories for the analysis of the tests carried out;
- number of satisfactory samples, i.e. the number of tests approved and paid for by the Ministry of Health;
- number of rejected tests and unsatisfactory samples.

The cytopathology test coverage indicator is one of the seven performance payment indicators of the Previne Brasil Program. Data was also collected for the cytopathology test coverage indicator from SISAB, organized by health unit and by four-month period.

### Data Analysis Procedures

As an estimate, the number of tests requested and registered was correlated with the monthly number of cytopathological tests in the process indicator of the Goal Record System, agreed upon by the States, municipalities, Federal District, and health regions and with the coverage of cytopathological tests registered in SISAB, in order to identify discrepancies between the number of tests registered by the health units and the number of tests actually registered in the Ministry of Health’s system, in order to check for under-registration.

In order to make the indicators comparable, the quantities of cytopathology tests recorded in the outsourced laboratories’ reports were added up by the corresponding months and grouped by four-month period, since the numerator of SISAB’s cytopathology test coverage indicator is available by four-month period on TabNet, which made it possible to estimate what we considered to be under-registrations. The financial value assigned was estimated by dividing the total amount paid by the municipality for the samples sent to the laboratories providing the service by the total number of tests, arriving at a unit value of R$ 4.01 per test. This value was multiplied by the total number of unregistered tests, considered under-registered.

Statistical analyses were carried out using the “R” statistical software, version 4.2.2.

### Ethical Aspects

This study was conducted in accordance with the recommendations for research with human beings issued by the National Health Council and was approved by the Ethics Committee of the Instituto Oswaldo Cruz, under opinion No. 5543104.

## RESULTS

In the last three years (2020, 2021, and 2022), 54,317 cytopathology tests were carried out at PA 3.1 health units. Of this total, 50.5%, or 27,424 tests, were carried out in 2022 alone. This figure reflects an increase of 75.2% in the last year (Table 1).

**Table 1:** Number of tests registered and paid for by the Unified Health System, carried out in outsourced laboratories in 2020, 2021, and 2022.

Unit	Registered and approved tests 2020	Registered and approved tests 2021	Registered and approved tests 2022	% of total tests carried out in 2022 by basic health unit	2022 growth compared to 2021
CF 01	159	386	987	3.6	155.70%
CF 02	351	471	810	3.0	72.00%
CF 03	309	468	852	3.1	82.10%
CF 04	58	239	711	2.6	197.50%
CF 05	291	460	796	2.9	73.00%
CF 06	345	280	587	2.1	109.60%
CF 07	780	1,109	1,905	6.9	71.80%
CF 08	363	517	626	2.3	21.10%
CF 09	237	307	815	3.0	165.50%
CF 10	274	394	856	3.1	117.30%
CF 11	204	652	1,037	3.8	59.00%
CF 12	265	235	987	3.6	320.00%
CF 13	369	246	896	3.3	264.20%
CF 14	73	263	585	2.1	122.40%
CF 15	388	357	925	3.4	159.10%
CF 16	251	523	896	3.3	71.30%
CF 17	390	560	696	2.5	24.30%
CF 18	1,783	1,937	2,183	8.0	12.70%
CMS 01	278	645	713	2.6	10.50%
CMS 02	66	184	258	0.9	40.20%
CMS 03	101	182	259	0.9	42.30%
CMS 04	467	513	972	3.5	89.50%
CMS 05	625	754	1,327	4.8	76.00%
CMS 06	302	716	838	3.1	17.00%
CMS 07	487	761	1,199	4.4	57.60%
CMS 08	504	718	1,065	3.9	48.30%
CMS 09	261	230	780	2.8	239.10%
CMS 10	577	681	1,184	4.3	73.90%
CMS 11	153	192	355	1.3	84.90%
CMS 12	372	85	475	1.7	458.80%
CMS 13	156	589	849	3.1	44.10%
**Grand total**	**11,239**	**15,654**	**27,424**	**100**	**75.20%**

CF: Family Clinic; CMS: Municipal Health Center.

Source: outsourced laboratory - *Associação Fundo de Incentivo à Pesquisa* (AFIP); reports from Municipal Health Secretariat (SMS - Rio).

Table 2 shows the number of tests carried out and registered by the health units in PA 3.1 and the number of tests for SISAB’s cytopathology coverage indicator per four-month period for the years 2021 and 2022, which made it possible to estimate the number of under-registrations for each of the health units in PA 3.1.

**Table 2:** Number of tests for the numerator of the cytopathology coverage indicator registered by PA 3.1 health unit in SISAB *versus* tests approved and paid for by the Unified Health System to outsourced laboratories in the four-month periods of 2021 and 2022.

Year	2021						2022					
Unit	Q. 1 – January to April		Q. 2 – May to August		Q. 3 – September to December		Q. 1 – January to April		Q. 2 – May to August		Q. 3 – September to December	
Registered cytopathological tests	Cytopathology coverage SISAB	Outsourced laboratories	Cytopathology coverage SISAB	Outsourced laboratories	Cytopathology coverage SISAB	Outsourced laboratories	Cytopathology coverage SISAB	Outsourced laboratories	Cytopathology coverage SISAB	Outsourced laboratories	Cytopathology coverage SISAB	Outsourced laboratories
CF 01	607	16	460	44	555	326	631	189	787	327	1,022	471
CF 02	1,270	56	996	234	804	181	857	190	888	267	1,052	353
CF 03	465	43	397	121	471	304	491	205	614	323	801	324
CF 04	592	10	519	28	515	201	547	75	622	243	790	393
CF 05	832	38	922	209	824	213	941	251	891	326	873	219
CF 06	785	28	654	14	635	238	675	97	638	187	724	303
CF 07	2,102	86	1,955	300	1,986	723	2,161	374	2,115	612	2,004	919
CF 08	596	34	582	123	694	360	795	173	818	189	937	264
CF 09	572	77	600	91	577	139	637	130	839	417	911	268
CF 10	644	18	599	71	601	305	697	206	714	280	705	370
CF 11	294	21	287	192	351	439	476	224	665	356	971	457
CF 12	867	24	660	39	672	172	768	167	788	279	1,065	541
CF 13	837	25	724	14	702	207	721	143	827	377	1,041	376
CF 14	438	16	360	20	472	227	536	171	586	247	613	167
CF 15	898	20	730	37	748	300	871	235	872	284	980	406
CF 16	520	84	506	140	583	299	715	282	726	255	814	359
CF 17	558	42	431	98	547	420	645	139	773	239	856	318
CF 18	2,584	289	2,405	620	2,556	1,028	2,832	638	2,747	738	2,728	807
CMS 01	617	92	405	301	335	252	422	214	526	194	689	305
CMS 02	349	23	289	49	288	112	296	45	329	114	359	99
CMS 03	31	5	103	87	177	90	215	43	291	83	409	133
CMS 04	884	90	809	196	894	227	1,059	309	1,091	280	1,090	383
CMS 05	543	160	557	187	573	407	695	372	731	442	1,004	513
CMS 06	596	122	629	339	677	255	715	129	694	250	871	459
CMS 07	1,102	26	1,023	285	1,098	450	1,272	419	1,276	382	1,361	398
CMS 08	1,066	60	861	172	886	486	884	126	887	344	1,015	595
CMS 09	389	8	393	84	424	138	447	94	466	200	800	486
CMS 10	874	97	734	231	638	353	635	178	654	307	1,134	699
CMS 11	605	45	488	85	413	62	384	2	328	85	465	268
CMS 12	682	25	616	6	526	54	541	142	498	172	456	161
CMS 13	449	87	358	140	551	362	646	173	746	245	856	431
CMS 14	1,126	0	1,143	0	1,181	0	1,144	157	1,125	355	1,140	261
**Grand total**	**24,774**	**1,767**	**22,195**	**4,557**	**22,954**	**9,330**	**25,351**	**6,292**	**26,552**	**9,399**	**30,536**	**12,506**

PA: programmatic area; CF: Family Clinic; CMS: Municipal Health Center; SMS: Municipal Health Secretariat; Fiocruz: *Fundação Oswaldo Cruz*; ENSP: *Escola Nacional de Saúde Pública*; CSE: School Health Center.

Source: outsourced laboratories; reports from Municipal Health Secretariat (SMS- Rio); SISAB: *Sistema de Informação em Saúde para a Atenção Básica* (Health Information System for Primary Care).

Table 3 shows the estimated under-registrations for each health unit in the years 2021 and 2022, with a total of 108,511 tests not registered in the information systems over the last two years in the entire PA 3.1 area. This corresponds to an estimated total of R$ 435,129.00 that would have been foregone by the municipality of Rio de Janeiro if the Previne Brasil Program had been in place during the period studied.

**Table 3:** Number of cytopathology tests that were no longer registered (under-registration) per health unit in PA 3.1 and their respective financial losses in 2021 and 2022.

Unit	2021						2022					
	Q, 1 – January to April		Q, 2 – May to August		Q, 3 – September to December		Q, 1 – January to April		Q, 2 – May to August		Q, 3 – September to December	
	Unregistered tests	Value lost due to under-registration	Unregistered tests	Value lost due to under-registration	Unregistered tests	Value lost due to under-registration	Unregistered tests	Value lost due to under-registration	Unregistered tests	Value lost due to under-registration	Unregistered tests	Value lost due to under-registration
CF 01	591	2,370	416	1,668	229	918	442	1,772	460	1,845	551	2,210
CF 02	1,214	4,868	762	3,056	623	2,498	667	2,675	621	2,490	699	2,803
CF 03	422	1,692	276	1,107	167	670	286	1,147	291	1,167	477	1,913
CF 04	582	2,334	491	1,969	314	1,259	472	1,893	379	1,520	397	1,592
CF 05	794	3,184	713	2,859	611	2,450	690	2,767	565	2,266	654	2,623
CF 06	757	3,036	640	2,566	397	1,592	578	2,318	451	1,809	421	1,688
CF 07	2,016	8,084	1,655	6,637	1,263	5,065	1,787	7,166	1,503	6,027	1,085	4,351
CF 08	562	2,254	459	1,841	334	1,339	622	2,494	629	2,522	673	2,699
CF 09	495	1,985	509	2,041	438	1,756	507	2,033	422	1,692	643	2,578
CF 10	626	2,510	528	2,117	296	1,187	491	1,969	434	1,740	335	1,343
CF 11	273	1,095	95	381	-88	-353	252	1,011	309	1,239	514	2,061
CF 12	843	3,380	621	2,490	500	2,005	601	2,410	509	2,041	524	2,101
CF 13	812	3,256	710	2,847	495	1,985	578	2,318	450	1,805	665	2,667
CF 14	422	1,692	340	1,363	245	982	365	1,464	339	1,359	446	1,788
CF 15	878	3,521	693	2,779	448	1,796	636	2,550	588	2,358	574	2,302
CF 16	436	1,748	366	1,468	284	1,139	433	1,736	471	1,889	455	1,825
CF 17	516	2,069	333	1,335	127	509	506	2,029	534	2,141	538	2,157
CF 18	2,295	9,203	1,785	7,158	1,528	6,127	2,194	8,798	2,009	8,056	1,921	7,703
CMS 01	525	2,105	104	417	83	333	208	834	332	1,331	384	1,540
CMS 02	326	1,307	240	962	176	706	251	1,007	215	862	260	1,043
CMS 03	26	104	16	64	87	349	172	690	208	834	276	1,107
CMS 04	794	3,184	613	2,458	667	2,675	750	3,008	811	3,252	707	2,835
CMS 05	383	1,536	370	1,484	166	666	323	1,295	289	1,159	491	1,969
CMS 06	474	1,901	290	1,163	422	1,692	586	2,350	444	1,780	412	1,652
CMS 07	1,076	4,315	738	2,959	648	2,598	853	3,421	894	3,585	963	3,862
CMS 08	1,006	4,034	689	2,763	400	1,604	758	3,040	543	2,177	420	1,684
CMS 09	381	1,528	309	1,239	286	1,147	353	1,416	266	1,067	314	1,259
CMS 10	777	3,116	503	2,017	285	1,143	457	1,833	347	1,391	435	1,744
CMS 11	560	2,246	403	1,616	351	1,408	382	1,532	243	974	197	790
CMS 12	657	2,635	610	2,446	472	1,893	399	1,600	326	1,307	295	1,183
CMS 13	362	1,452	218	874	189	758	473	1,897	501	2,009	425	1,704
CMS 14	1,126	4,515	1,143	4,583	1,181	4,736	987	3,958	770	3,088	879	3,525
**Grand total**	**23,007**	**92,258**	**17,638**	**70,728**	**13,624**	**54,632**	**19,059**	**76,427**	**17,153**	**68,784**	**18,030**	**72,300**

PA: programmatic area; CF: Family Clinic; CMS: Municipal Health Center; SMS: Municipal Health Secretariat; Fiocruz: *Fundação Oswaldo Cruz*; ENSP: *Escola Nacional de Saúde Pública*; CSE: School Health Center.

Note: under-registration as the difference between the number of tests recorded in the numerator of the vaccination coverage indicator in the Primary Health Care Information System and the number of cytopathology tests paid for by the Unified Health System to third-party laboratories.

## DISCUSSION

From the perspective of PHC, as the main gateway and care coordinator for health care networks, the effects of under-reporting extend to all levels of care, from the lack of records in medical records (physical or electronic) to the under-reporting of diseases and illnesses in the Notifiable Diseases Information System (SINAN), which is a challenge for managers^12,13^. In this sense, the method used to quantify under-reporting is important for sizing up the problem, especially as it potentially delves into the historical under-funding of the SUS.

The findings of this study show that there are difficulties inherent in local management processes when dealing with sudden increases in test authorizations, as occurred in November 2021 and 2022. The vulnerable territories analyzed had administrative difficulties in registering people, indicating that cities with large population clusters in less developed areas tend to have fewer resources and greater difficulty in registration. In addition, the period of this study encompasses the resumption of repressed demand due to the COVID-19 pandemic, which in itself represented a significant stress for the ordering of care, increasing the relevance of the potential losses caused by under-registration.

It should also be pointed out that any changes to the *modus operandi* are always met with resistance, as well as requiring changes to work processes. In this sense, the recording of user information - especially if it involves the implementation of new medical records models - involves a complex process, with the participation of health professionals in various dimensions: technical, human, individual, and organizational^14^.

In the case of computerized records, which are increasingly being adopted, the usability aspects of the software cannot be disregarded, as they interfere with adherence, efficiency, and, consequently, the quality, completeness, and reliability of the record^15^.

A resilient health system must be able to adapt to emerging needs but also sustain the functioning of its regular, problem-solving services at adequate levels of quality, even in times of crisis. The WHO (2010) places funding as one of the so-called “building blocks” of resilient health systems^16,17^ in that an adequate budget is indispensable to the ability of health systems to maintain and continuously improve their essential functions. However, financing is much more than a simple allocation of resources. Understanding the nature of the indicators that can be used to monitor and evaluate the financing of health systems requires a specific assessment of what is hoped to be achieved^16,18^.

Developing the system’s resilience involves ensuring the availability of resources to deal with the dynamics of the territory, with seasonal transitions that cause fluctuations in population coverage of various programs, as well as susceptibility to adverse events of different natures and intensities.

As the findings of this study show, the losses generated by under-registration jeopardize the realization of fundamental investments for the development of resilient skills in territories whose dynamics demand continuous capacity for adaptation, prevention, and absorption of shocks, such as ensuring an adequate number of trained health personnel, infrastructure and services and other elements of institutional capacity essential for coping with short and medium-term risks^19^.

Changes to the PHC intergovernmental transfer model come up against technical, human, individual, and organizational obstacles that affect how user information is recorded^14^. According to Massuda^7^ and Costa et al.^3^, this new PHC financing policy will have a number of impacts on the resilience and sustainability of the SUS and on the health of the population that need to be identified and monitored over the next few years, especially given the long-term maintenance of fiscal austerity measures, which are likely to aggravate public health underfunding in the country.

The estimated increase in the volume of under-registration in the area analyzed also reveals the weakening resilience of SUS services in the region, which could be aggravated if the reduction in financial transfers is confirmed. As the ability to monitor and anticipate short-term disturbances is weakened, resolutiveness at the PHC level in times of crisis becomes even more of a determining factor in the pressure on other levels of care. Therefore, when looking at the growing estimates shown in Table 1, it is important to highlight the need for a transition process in the financing model that considers the characteristics of each territory, especially the way in which vulnerability interferes with the management mechanisms of health units.

Especially in crisis scenarios, government intervention should focus on mitigating, not provoking, economic contraction. More specifically, a good health policy - or change in existing health policy - should maintain the flow of resources needed to sustain services in line with fluctuating demand, as was done until the last quarter of 2022, when the Previne Brasil Program, although adopted, had not yet made pay for performance effective^20,21^.

In general, health spending should adopt a countercyclical logic in times of crisis, since the fundamental objective of a public policy is to guarantee the population - especially the poorest and most vulnerable - access to essential services, and this can only be achieved by preserving health sector funding from the effects of shocks.

A resilient response by health systems to a shock involves seeking strategies that ensure the performance of health system functions in a sustainable manner, which means protecting the overall functioning of the system from budgetary constraints. In addition, changes in resource transfer policies must not compromise the planned provision and operation of services, nor their capacity to be adjusted according to the reality of the territories, at the risk of also compromising the overall performance of the system and, by extension, its resilience^22^.

The Previne Brasil Program has been the subject of exhaustive debate, pointing out its privatizing, selective, and focused nature, in the opposite direction to the initial proposals for the Family Health Strategy in the National Primary Care Policy (PNAB) of 2006^23^ and 2012^24^, which were moving towards universal coverage and access, in line with the global agenda advocated by organizations such as the World Health Organization (WHO) and the United Nations^20,25^.

The findings of this study show that, in terms of remuneration for performance, territories with the characteristics of PA 3.1 in the municipality of Rio de Janeiro would lose, in estimated values, more than R$ 430,000.00 due to under-registration. The figures found show significant losses equivalent to the funding coverage of 133 40-hour family health teams, 177 30-hour family health teams, or 266 20-hour family health teams - figures which, in a historical context of underfunding, should not be overlooked.

Chioro et al.^26^ reflect on the changes in public policies due to the ideological profile of governments. The resilience and sustainability of the SUS depends fundamentally on democracy and public support for the system, which needs to be increasingly effective, even when dealing with limited resources. On the other hand, as highlighted by the WHO^27^, the introduction of reforms to the way services are paid for can have unexpected and negative consequences. It is therefore recommended that changes be incremental and avoid sudden ruptures that could cause side effects for the health system.

In this sense, although it is hoped that the Previne Brasil Program can stimulate improvements in registration processes and that weighting will value more vulnerable regions, this study indicates that funding will be impacted by the current state of effectiveness of the registry and should vary substantially across the country^28,29^.

## CONCLUSION

The main contribution of this article lies in the presentation of empirical evidence of the potential effects of under-registration on PHC financing. In addition, there are two other significant findings - firstly, it highlights weaknesses in the process of recording health information inherent to vulnerable regions; secondly, it indicates a vicious circle potentially fueled by sudden changes in PHC funding conditions, as well as potential consequences for other levels of care.

Regarding the various dimensions that affect the registration process, this study is limited to the information available on the DATASUS database, which essentially refers to institutional capacity and reveals little about the behavior of the services. In this sense, future in-depth studies will be pertinent in order to concretely highlight the causes of the disturbances in the work of collecting and analyzing health information in the territories.

It is important to note that further research should be carried out on the financial impacts that the new form of financing primary care may have, especially regarding a larger scale, i.e. including more municipalities, as well as the other procedures and indicators that are part of the Previne Brasil Program indicators.
