# Supplementary material for: Effects of pay for performance in primary care in an under-registration scenario
Source: Rev Saude Publica. 2024 Oct 4;58:44. doi: 10.11606/s1518-8787.2024058005812 (PMC11548914; doi:10.11606/s1518-8787.2024058005812)
Supplement: Translation

# Translation: Efeitos da remuneração por desempenho na atenção primária em um cenário de sub-registro

**Authors:** Paula de Castro-Nunes, Paloma Palmieri, Hugo Bellas, Adriana Soares, Jaqueline Viana, Paulo Victor Rodrigues de Carvalho, Alessandro Jatobá

**Affiliations:** I Brasil Fundação Oswaldo Cruz. Centro de Estudos Estratégicos Antônio Ivo de Carvalho. Rio de Janeiro, RJ, Brasil; II Brasil Secretaria Municipal de Saúde do Rio de Janeiro. Rio de Janeiro, RJ, Brasil

**Keywords:** Sub-registro, Financiamento da Assistência à Saúde, Financiamento dos Sistemas de Saúde

## Abstract

**OBJETIVO:**

Propor um método de detecção e análise do sub-registro e evidenciar o seu potencial efeito financeiro em face da implementação do Programa Previne Brasil.

**MÉTODOS:**

Foi realizado um estudo ecológico de análise dos exames citopatológicos na área programática 3.1 do município do Rio de Janeiro. Os dados foram coletados do barramento do Datasus, incluindo as informações relativas aos relatórios dos laboratórios terceirizados dos exames citopatológicos e aquelas disponíveis no Sistema de Informação em Saúde para a Atenção Básica (Sisab) e Sistema de Informação do Câncer do Colo do Útero (Siscolo) do Datasus/Ministério da Saúde.

**RESULTADOS:**

Os sub-registros estimados por unidade de saúde totalizaram 108.511 exames nos últimos dois anos na área programática 3.1, o que corresponde a um total estimado de R$ 435.129,00 que teriam sido deixados de receber, caso o Programa Previne Brasil já estivesse efetivado no período estudado.

**CONCLUSÃO:**

A principal contribuição do artigo está na apresentação de evidência empírica dos potenciais efeitos do sub-registro para o financiamento da atenção primária à saúde. Além disso, há dois outros achados significativos – primeiro, evidencia fragilidades no processo de registro das informações de saúde inerentes a regiões vulneráveis; segundo, indica um círculo vicioso potencialmente alimentado pela alteração brusca dos condicionantes do financiamento da atenção primária à saúde, além de potenciais consequências para os demais níveis de atenção.

## INTRODUÇÃO

O Programa Previne Brasil, instituído pela Portaria n^o^ 2.979 de 12 de novembro de 2019^1^, alterou as formas de repasse de recursos para o financiamento da atenção primária à saúde (APS), que passaram a ser distribuídos com base em quatro critérios: captação ponderada; pagamento por desempenho; incentivo para ações estratégicas; e incentivo financeiro com base em critério populacional. Em relação ao modelo anterior de financiamento, destaca-se que o repasse com base no desempenho dos serviços substituiu o piso da atenção básica (PAB) fixo, que garantia repasses mínimos com base nas populações adscritas. Esta mudança não tensionou apenas a cobertura da APS, pois passou a demandar mecanismos de gestão para garantir a confiabilidade do registro dos procedimentos efetuados pelas secretarias de saúde. Isso é um problema para os territórios mais vulneráveis, nos quais o registro das informações de saúde sofre diferentes perturbações, desde as dificuldades de captação até a falta de recursos para a análise adequada.

Antes da instituição do Programa Previne Brasil, o financiamento da atenção primária era composto pelos PAB fixo e variável. O valor do PAB fixo era calculado *per capita,* ou seja, com base na população estimada pelo Instituto Brasileiro de Geografia e Estatística (IBGE) em relação à população adscrita, variando de R$ 23,00 a R$ 28,00. Já o PAB variável era condicionado à implementação da Estratégia de Saúde da Família pelos gestores municipais^2^. Desta forma, a introdução do Previne Brasil alterou o financiamento das equipes, o que implicou adotar novos procedimentos de alimentação dos dados e, consequentemente, novas rotinas e processos de gestão para os profissionais da APS^3^.

É importante assinalar que os registros referentes aos procedimentos, exames, consultas e condutas adotadas pelos profissionais de saúde possibilitam a comunicação entre membros da equipe multidisciplinar e a continuidade e longitudinalidade da assistência prestada ao usuário. Ao fornecer evidências diretas dos eventos de saúde dos territórios, subsidiam os processos de tomada de decisão, planejamento, execução e avaliação. São, portanto, parâmetros primários para a remuneração por desempenho, mesmo não sendo indicadores precisos da expansão do acesso ou da qualidade dos serviços. Além disso, a falta ou incompletude do registro das informações relativas ao perfil socioeconômico, à situação de saúde dos pacientes e os procedimentos realizados durante os atendimentos é um problema prevalente e histórico no Sistema Único de Saúde (SUS)^4^.

O sub-registro refere-se à quantidade de eventos que deveriam ser registrados, mas acabam ignorados ou perdidos nos sistemas de informação em saúde. Isso pode ocorrer em diversos âmbitos, tais como: comunidade, centros de trabalho, serviços de saúde e Sistema de Vigilância Epidemiológica, entre outros^8^.

A introdução de um modelo de financiamento baseado no desempenho – especialmente com a descontinuidade do PAB fixo – aumenta o potencial para o recrudescimento do subfinanciamento, especialmente em regiões ou municípios mais vulneráreis, que usualmente enfrentam mais dificuldades para modernizar seus processos de gestão, no sentido de garantir a confiabilidade do registro^3^.

Desse modo, compreender os impactos financeiros do sub-registro na alocação de recursos da APS e informar aos agentes envolvidos sobre a necessidade de melhoria de sua gestão passa a ser de fundamental importância para viabilizar e garantir o sucesso da implantação de novos modelos de pagamento por desempenho no SUS. Nesse contexto, o artigo evidencia o potencial efeito financeiro do sub-registro em face da implementação do Programa Previne Brasil, propondo um método de análise em que se analisa os exames citopatológicos na área programática (AP) 3.1 do município do Rio de Janeiro.

A linha de cuidado de saúde da mulher, diretamente relacionada à demanda por exames citopatológicos, foi escolhida para este estudo por ser representativa da importância de informação qualificada na articulação entre os níveis de atenção para a resiliência dos serviços. Seu registro correto importa não apenas em função de eventuais impactos financeiros, mas também na medida em que a capacidade de resposta dos serviços a agravos como o câncer do colo do útero é dimensionada considerando análises dos registros feitos no nível da APS. Segundo dados do Instituto Nacional de Câncer (INCA)^9,10^, no Brasil, com exceção dos tumores de pele não melanoma, o câncer do colo do útero é o terceiro tipo de câncer mais incidente entre as mulheres. Somente para o ano de 2023, foram estimados 17.010 novos casos.

## MÉTODOS

### Desenho da Pesquisa

Trata-se de um estudo observacional ecológico, de natureza quantitativa, que tem como fontes de informação os dados dos sistemas de gestão da Secretaria Municipal de Saúde do Rio de Janeiro (SMS-Rio), do barramento de dados do Departamento de Informática do Sistema Único de Saúde (Datasus) e de laboratórios terceirizados que prestam serviços ao Ministério da Saúde da Associação Fundo de Incentivo à Pesquisa (AFIP). Segundo os procedimentos de coleta, o estudo foi situado na AP 3.1 do município do Rio de Janeiro.

Este artigo foi elaborado de acordo com as recomendações das diretrizes da EQUATOR Network, a lista de checagem *Strengthening the Reporting of Observational Studies in Epidemiology* (STROBE)^11^.

### Ambiente de Pesquisa

A rede de assistência do município do Rio de Janeiro é distribuída entre oito Coordenadorias de Áreas Programáticas (CAPs), cobrindo todo o território do município (que atende aproximadamente 6.211.423 habitantes, segundo dados do IBGE), a saber: CAP 1.0 (região central); CAP 2.1 (bairros da zona sul); CAP 3.1, CAP 3.2 e CAP 3.3 (bairros da zona norte); CAP 4.0, CAP 5.2 e CAP 5.3 (zona oeste).

A pesquisa foi desenvolvida na CAP 3.1, escolhida por ser representativa do conjunto e por refletir o contexto sanitário e de vulnerabilidade do município do Rio de Janeiro. Além disso, esta AP conta com 32 unidades de saúde e o maior número de equipes (218), possui grandes áreas de vulnerabilidade social, econômica e ambiental e desigualdade social, já que engloba três grandes complexos de favelas. A AP 3.1 abrange uma área de 85,36 km^2^, com densidade demográfica de 10.386 habitantes/km^2^, e possui 886.551 residentes, dos quais, 80% com cobertura da Estratégia de Saúde da Família, num total de 714.598 cadastrados.

Entre as áreas cobertas pela CAP 3.1, identifica-se uma desigualdade latente, exemplificada pelo Jardim Guanabara – com 0,963 de índice de desenvolvimento humano (IDH), em terceiro lugar entre os 126 bairros do município do Rio de Janeiro – e o Complexo do Alemão, em 126^o^ lugar (último) com 0,711. Ao analisar o índice de desenvolvimento social (IDS) – que contempla as dimensões saneamento básico, serviço de coleta de lixo, banheiro para moradores, analfabetismo e renda média –, a região apresenta um índice de 0,518, abrangendo as áreas mais vulneráveis da cidade.

### Fontes de Informação

Os dados utilizados neste estudo foram coletados do sistema de gestão da CAP 3.1 e da SMS-Rio e também são de acesso livre por meio do barramento de dados do Datasus e do laboratório terceirizado prestador de serviços (AFIP). Foram incluídas as informações relativas ao processo de avaliação, controle, regulação do planejamento assistencial e gestão financeira, como os relatórios dos laboratórios terceirizados relativos aos exames citopatológicos e aqueles disponíveis no Sistema de Informação em Saúde para a Atenção Básica (Sisab) e no Sistema de Informação do Câncer do Colo do Útero (Siscolo) do Datasus/Ministério da Saúde, além de dados coletados diretamente do TabNet da SMS-Rio.

### Procedimento de Coleta de Dados

Coletou-se dados referentes ao número de exames citopatológicos realizados pelas 32 unidades de APS sob a gestão da CAP 3.1 da SMS-Rio, no departamento de informações. Os dados referem-se a:

- número de exames solicitados e registrados de pacientes do sexo feminino de 25 a 64 anos de idade residentes no município do Rio de Janeiro, organizados por unidade de saúde, mês e ano;
- valores pagos aos laboratórios terceirizados pelas análises dos exames realizados;
•número de amostras satisfatórias, isto é, a quantidade de exames aprovados e pagos pelo Ministério da Saúde;
- número de exames rejeitados e com amostras insatisfatórias.

O indicador de cobertura de exames citopatológicos consiste em um dos sete indicadores para pagamento por desempenho do Programa Previne Brasil. Foram coletados também dados do indicador de cobertura de exames citopatológicos do Sisab, organizados por unidade de saúde e por quadrimestre.

### Procedimentos de Análise de Dados

Como forma de estimativa, o número de exames solicitados e registrados foi correlacionado ao número mensal de exames citopatológicos do indicador de processo do Sistema de Registro das Metas Pactuadas por Municípios, Regiões de Saúde, Estados e Distrito Federal e com a cobertura do exame citopatológico registrada no Sisab, de forma a identificar as discrepâncias entre o número de exames registrados pelas unidades de saúde e o número de exames efetivamente registrados no sistema do Ministério da Saúde, a fim de verificar o sub-registro.

Para que os indicadores se tornassem comparáveis, as quantidades dos exames citopatológicos registrados nos relatórios dos laboratórios terceirizados foram somadas pelos meses correspondentes e agrupadas por quadrimestre, uma vez que o numerador do indicador de cobertura dos exames citopatológicos do Sisab estão disponíveis por quadrimestre no TabNet, o que possibilitou a estimação do que consideramos sub-registros. O valor financeiro atribuído foi estimado dividindo-se o valor total pago pelo município pelas amostras enviadas aos laboratórios provedores do serviço pelo número total de exames, chegando ao valor unitário de R$ 4,01 por exame. Este valor foi multiplicado pelo número total de exames não registrados, considerados sub-registrados.

As análises estatísticas foram realizadas com o apoio do *software* estatístico “R”, versão 4.2.2.

### Aspectos Éticos

O presente estudo foi conduzido de acordo com as recomendações para pesquisa com seres humanos expedidas pelo Conselho Nacional de Saúde, tendo sido aprovado pelo Comitê de Ética do Instituto Oswaldo Cruz, por meio do parecer de n^o^ 5543104. Destaca-se que os nomes das unidades foram modificados.

## RESULTADOS

Foram realizados 54.317 exames citopatológicos nas unidades de saúde da AP 3.1 nos últimos três anos (2020, 2021 e 2022). Deste total, 50,5%, ou seja, 27.424 exames, foram realizados apenas no ano de 2022. Este quantitativo reflete um crescimento de 75,2% no último ano (Tabela 1).

**Tabela 1:** Número de exames registrados e pagos pelo Sistema Único de Saúde, realizados em laboratórios terceirizados nos anos de 2020, 2021 e 2022.

Unidade	Exames registrados e aprovados 2020	Exames registrados e aprovados 2021	Exames registrados e aprovados 2022	% do total de exames realizados em 2022 por unidade básica de saúde	Crescimento de 2022 em relação ao ano de 2021
CF 01	159	386	987	3,6	155,70%
CF 02	351	471	810	3,0	72,00%
CF 03	309	468	852	3,1	82,10%
CF 04	58	239	711	2,6	197,50%
CF 05	291	460	796	2,9	73,00%
CF 06	345	280	587	2,1	109,60%
CF 07	780	1.109	1.905	6,9	71,80%
CF 08	363	517	626	2,3	21,10%
CF 09	237	307	815	3,0	165,50%
CF 10	274	394	856	3,1	117,30%
CF 11	204	652	1.037	3,8	59,00%
CF 12	265	235	987	3,6	320,00%
CF 13	369	246	896	3,3	264,20%
CF 14	73	263	585	2,1	122,40%
CF 15	388	357	925	3,4	159,10%
CF 16	251	523	896	3,3	71,30%
CF 17	390	560	696	2,5	24,30%
CF 18	1.783	1.937	2.183	8,0	12,70%
CMS 01	278	645	713	2,6	10,50%
CMS 02	66	184	258	0,9	40,20%
CMS 03	101	182	259	0,9	42,30%
CMS 04	467	513	972	3,5	89,50%
CMS 05	625	754	1.327	4,8	76,00%
CMS 06	302	716	838	3,1	17,00%
CMS 07	487	761	1.199	4,4	57,60%
CMS 08	504	718	1.065	3,9	48,30%
CMS 09	261	230	780	2,8	239,10%
CMS 10	577	681	1.184	4,3	73,90%
CMS 11	153	192	355	1,3	84,90%
CMS 12	372	85	475	1,7	458,80%
CMS 13	156	589	849	3,1	44,10%
**Total geral**	**11.239**	**15.654**	**27.424**	**100**	**75,20%**

CF: Clínica da Família; CMS: Centro Municipal de Saúde.

Fonte: Laboratório terceirizado - Associação Fundo de Incentivo à Pesquisa (AFIP); Relatórios da Secretária Municipal de Saúde do Rio de Janeiro (SMS-Rio).

Tabela 2 apresenta os quantitativos dos exames realizados e registrados pelas unidades de saúde da AP 3.1 e o número de exames do indicador de cobertura de citopatológicos do Sisab por quadrimestre para os anos de 2021 e 2022, o que permitiu realizar a estimação do número de sub-registros para cada uma das unidades de saúde da AP 3.1.

**Tabela 2:** Número de exames do numerador do indicador de cobertura de citopatológico registrado por unidade de saúde da AP 3.1 no Sisab *versus* exames aprovados e pagos pelo Sistema Único de Saúde aos laboratórios terceirizados nos quadrimestres dos anos de 2021 e 2022.

Ano	2021						2022					
Unidade	Q. 1 – janeiro a abril		Q. 2 – maio a agosto		Q. 3 – setembro a dezembro		Q. 1 – janeiro a abril		Q. 2 – maio a agosto		Q. 3 – setembro a dezembro	
Exames citopatológicos registrados	Cobertura de citopatológico Sisab	Laboratórios terceirizados	Cobertura de citopatológico Sisab	Laboratórios terceirizados	Cobertura de citopatológico Sisab	Laboratórios terceirizados	Cobertura de citopatológico Sisab	Laboratórios terceirizados	Cobertura de citopatológico Sisab	Laboratórios terceirizados	Cobertura de citopatológico Sisab	Laboratórios terceirizados
CF 01	607	16	460	44	555	326	631	189	787	327	1.022	471
CF 02	1.270	56	996	234	804	181	857	190	888	267	1.052	353
CF 03	465	43	397	121	471	304	491	205	614	323	801	324
CF 04	592	10	519	28	515	201	547	75	622	243	790	393
CF 05	832	38	922	209	824	213	941	251	891	326	873	219
CF 06	785	28	654	14	635	238	675	97	638	187	724	303
CF 07	2.102	86	1.955	300	1.986	723	2.161	374	2.115	612	2.004	919
CF 08	596	34	582	123	694	360	795	173	818	189	937	264
CF 09	572	77	600	91	577	139	637	130	839	417	911	268
CF 10	644	18	599	71	601	305	697	206	714	280	705	370
CF 11	294	21	287	192	351	439	476	224	665	356	971	457
CF 12	867	24	660	39	672	172	768	167	788	279	1.065	541
CF 13	837	25	724	14	702	207	721	143	827	377	1.041	376
CF 14	438	16	360	20	472	227	536	171	586	247	613	167
CF 15	898	20	730	37	748	300	871	235	872	284	980	406
CF 16	520	84	506	140	583	299	715	282	726	255	814	359
CF 17	558	42	431	98	547	420	645	139	773	239	856	318
CF 18	2.584	289	2.405	620	2.556	1.028	2.832	638	2.747	738	2.728	807
CMS 01	617	92	405	301	335	252	422	214	526	194	689	305
CMS 02	349	23	289	49	288	112	296	45	329	114	359	99
CMS 03	31	5	103	87	177	90	215	43	291	83	409	133
CMS 04	884	90	809	196	894	227	1.059	309	1.091	280	1.090	383
CMS 05	543	160	557	187	573	407	695	372	731	442	1.004	513
CMS 06	596	122	629	339	677	255	715	129	694	250	871	459
CMS 07	1.102	26	1.023	285	1.098	450	1.272	419	1.276	382	1.361	398
CMS 08	1.066	60	861	172	886	486	884	126	887	344	1.015	595
CMS 09	389	8	393	84	424	138	447	94	466	200	800	486
CMS 10	874	97	734	231	638	353	635	178	654	307	1.134	699
CMS 11	605	45	488	85	413	62	384	2	328	85	465	268
CMS 12	682	25	616	6	526	54	541	142	498	172	456	161
CMS 13	449	87	358	140	551	362	646	173	746	245	856	431
CMS 14	1.126	0	1.143	0	1.181	0	1.144	157	1.125	355	1.140	261
**Total geral**	**24.774**	**1.767**	**22.195**	**4.557**	**22.954**	**9.330**	**25.351**	**6.292**	**26.552**	**9.399**	**30.536**	**12.506**

AP: área programática; CF: Clínica da Família; CMS: Centro Municipal de Saúde; SMS: Secretaria Municipal de Saúde; Fiocruz: Fundação Oswaldo Cruz; ENSP: Escola Nacional de Saúde Pública; CSE: Centro de Saúde Escola.

Fonte: laboratórios terceirizados; relatórios da Secretária Municipal da Saúde do Rio de Janeiro (SMS-Rio); Sisab: Sistema de Informação em Saúde para a Atenção Básica.

A Tabela 3 apresenta os sub-registros estimados para cada unidade de saúde nos anos de 2021 e 2022, com um total de 108.511 exames não registrados nos sistemas de informação nos últimos dois anos em toda a área da AP 3.1. Isso corresponde a um total estimado de R$ 435.129,00 que teriam sido deixados de receber pelo município do Rio de Janeiro, caso o Programa Previne Brasil já estivesse efetivado no período estudado.

**Tabela 3:** Quantidade de exames citopatológicos que deixaram de ser registrados (sub-registros) por unidade de saúde da AP 3.1 e suas respectivas perdas financeiras nos anos de 2021 e 2022.

Unidade	2021						2022					
	Q. 1 – janeiro a abril		Q. 2 – maio a agosto		Q. 3 – setembro a dezembro		Q. 1 – janeiro a abril		Q. 2 – maio a agosto		Q. 3 – setembro a dezembro	
	Exames não registrados	Valor perdido devido ao sub-registro	Exames não registrados	Valor perdido devido ao sub-registro	Exames não registrados	Valor perdido devido ao sub-registro	Exames não registrados	Valor perdido devido ao sub-registro	Exames não registrados	Valor perdido devido ao sub-registro	Exames não registrados	Valor perdido devido ao sub-registro
CF 01	591	2.370	416	1.668	229	918	442	1.772	460	1.845	551	2.210
CF 02	1.214	4.868	762	3.056	623	2.498	667	2.675	621	2.490	699	2.803
CF 03	422	1.692	276	1.107	167	670	286	1.147	291	1.167	477	1.913
CF 04	582	2.334	491	1.969	314	1.259	472	1.893	379	1.520	397	1.592
CF 05	794	3.184	713	2.859	611	2.450	690	2.767	565	2.266	654	2.623
CF 06	757	3.036	640	2.566	397	1.592	578	2.318	451	1.809	421	1.688
CF 07	2.016	8.084	1.655	6.637	1.263	5.065	1.787	7.166	1.503	6.027	1.085	4.351
CF 08	562	2.254	459	1.841	334	1.339	622	2.494	629	2.522	673	2.699
CF 09	495	1.985	509	2.041	438	1.756	507	2.033	422	1.692	643	2.578
CF 10	626	2.510	528	2.117	296	1.187	491	1.969	434	1.740	335	1.343
CF 11	273	1.095	95	381	-88	-353	252	1.011	309	1.239	514	2.061
CF 12	843	3.380	621	2.490	500	2.005	601	2.410	509	2.041	524	2.101
CF 13	812	3.256	710	2.847	495	1.985	578	2.318	450	1.805	665	2.667
CF 14	422	1.692	340	1.363	245	982	365	1.464	339	1.359	446	1.788
CF 15	878	3.521	693	2.779	448	1.796	636	2.550	588	2.358	574	2.302
CF 16	436	1.748	366	1.468	284	1.139	433	1.736	471	1.889	455	1.825
CF 17	516	2.069	333	1.335	127	509	506	2.029	534	2.141	538	2.157
CF 18	2.295	9.203	1.785	7.158	1.528	6.127	2.194	8.798	2.009	8.056	1.921	7.703
CMS 01	525	2.105	104	417	83	333	208	834	332	1.331	384	1.540
CMS 02	326	1.307	240	962	176	706	251	1.007	215	862	260	1.043
CMS 03	26	104	16	64	87	349	172	690	208	834	276	1.107
CMS 04	794	3.184	613	2.458	667	2.675	750	3.008	811	3.252	707	2.835
CMS 05	383	1.536	370	1.484	166	666	323	1.295	289	1.159	491	1.969
CMS 06	474	1.901	290	1.163	422	1.692	586	2.350	444	1.780	412	1.652
CMS 07	1.076	4.315	738	2.959	648	2.598	853	3.421	894	3.585	963	3.862
CMS 08	1.006	4.034	689	2.763	400	1.604	758	3.040	543	2.177	420	1.684
CMS 09	381	1.528	309	1.239	286	1.147	353	1.416	266	1.067	314	1.259
CMS 10	777	3.116	503	2.017	285	1.143	457	1.833	347	1.391	435	1.744
CMS 11	560	2.246	403	1.616	351	1.408	382	1.532	243	974	197	790
CMS 12	657	2.635	610	2.446	472	1.893	399	1.600	326	1.307	295	1.183
CMS 13	362	1.452	218	874	189	758	473	1.897	501	2.009	425	1.704
CMS 14	1.126	4.515	1.143	4.583	1.181	4.736	987	3.958	770	3.088	879	3.525
**Total geral**	**23.007**	**92.258**	**17.638**	**70.728**	**13.624**	**54.632**	**19.059**	**76.427**	**17.153**	**68.784**	**18.030**	**72.300**

AP: área programática; CF: Clínica da Família; CMS: Centro Municipal de Saúde; SMS: Secretaria Municipal de Saúde; Fiocruz: Fundação Oswaldo Cruz; ENSP: Escola Nacional de Saúde Pública; CSE: Centro de Saúde Escola.

Nota: sub-registros como sendo a diferença entre o número de exames registrados no numerador do indicador de cobertura vacinal do Sistema de Informação em Saúde para a Atenção Básica e as quantidades de exames citopatológicos pagos pelo Sistema Único de Saúde aos laboratórios terceirizados conveniados.

## DISCUSSÃO

Dentro da perspectiva da APS, enquanto principal porta de entrada e ordenadora do cuidado das redes de atenção em saúde, os efeitos do sub-registro se estendem por todos os níveis de atenção, desde a falta de registros em prontuários (físicos ou eletrônicos) até a subnotificação de doenças e agravos do Sistema Nacional de Notificação (Sinan), sendo um desafio para os gestores^12,13^. Nesse sentido, o método aplicado para a quantificação do sub-registro é importante para o dimensionamento do problema, principalmente por apresentar um potencial aprofundamento no histórico subfinanciamento do SUS.

Os achados deste estudo demonstram que há dificuldades inerentes aos processos locais de gestão ao lidar com aumentos repentinos de autorizações para exames, como ocorrido nos meses de novembro de 2021 e 2022. Os territórios vulneráveis analisados apresentaram dificuldade administrativa para registro das pessoas, indicando que cidades com grandes aglomerados populacionais em áreas menos desenvolvidas tendem a dispor de menos recursos e maior dificuldade no registro. Além disso, o período deste estudo engloba a retomada da demanda reprimida pela pandemia de covid-19, o que, em si, representou um estresse significativo para a ordenação do cuidado, aumentando a relevância das potenciais perdas provocadas pelo sub-registro.

Cabe destacar também que quaisquer rupturas no *modus operandi* sempre encontram resistência, além de exigir mudança nos processos de trabalho. Nesse sentido, o registro da informação dos usuários – especialmente se abranger a implementação de novos modelos de prontuários – envolve um processo complexo, com a participação dos profissionais de saúde em várias dimensões: técnicas, humanas, individuais e organizacionais^14^.

No caso de registros informatizados, cada vez mais adotados, não podem ser desconsiderados os aspectos de usabilidade do *software*, que interferem na adesão, eficiência e, consequentemente, na qualidade, completude e confiabilidade do registro^15^.

Um sistema de saúde resiliente deve ser capaz de se adaptar às necessidades emergentes, mas também sustentar o funcionamento de seus serviços regulares, resolutivos, em níveis adequados de qualidade, mesmo nos momentos de crise. A OMS (2010) coloca o financiamento como um dos chamados “blocos de construção” dos sistemas de saúde resilientes^16,17^, na medida em que um orçamento adequado é indispensável à capacidade dos sistemas de saúde de manter e melhorar continuamente suas funções essenciais. No entanto, o financiamento é muito mais do que uma simples alocação de recursos. Compreender a natureza dos indicadores que podem ser utilizados para monitorar e avaliar o financiamento dos sistemas de saúde requer uma avaliação específica do que se espera alcançar^16,18^.

Desenvolver a resiliência do sistema envolve garantir a disponibilidade de recursos para lidar com a dinâmica do território, com transições sazonais que provocam flutuações da cobertura populacional de programas diversos, além da suscetibilidade a eventos adversos de diferentes naturezas e intensidades.

Como os achados do presente estudo demonstram, as perdas geradas por sub-registro comprometem a realização de investimentos fundamentais para o desenvolvimento de habilidades resilientes em territórios cuja dinâmica demanda capacidade contínua de adaptação, prevenção e absorção de choques, como garantir um número adequado de pessoal de saúde treinado, infraestrutura e serviços e outros elementos da capacidade institucional essenciais para o enfrentamento de riscos de curto e médio prazos^19^.

As mudanças no modelo de transferências intergovernamentais da APS esbarram em obstáculos de natureza técnica, humana, individual e organizacional que afetam como o registro da informação dos usuários é realizado^14^. Segundo Massuda^7^ e Costa et al.^3^, essa nova política de financiamento da APS terá um conjunto de impactos para a resiliência e sustentabilidade do SUS e para a saúde da população que precisam ser identificados e monitorados ao longo dos próximos anos, principalmente diante da manutenção por longo prazo das medidas de austeridade fiscal, que deverá agravar o subfinanciamento público da saúde no país.

A estimativa de aumento do volume de sub-registro na área analisada também é reveladora do enfraquecimento da resiliência dos serviços do SUS na região, o que pode ser agravado caso se confirme a redução dos repasses financeiros. Como a capacidade de monitorar e antecipar perturbações de curto prazo está fragilizada, a resolutividade no nível da APS em tempos de crise torna-se ainda mais determinante da pressão sobre os demais níveis de atenção. Portanto, ao observar a estimativa crescente apresentada na Tabela 1, é importante destacar a necessidade de um processo de transição do modelo de financiamento que considere as características de cada território, em especial a forma pela qual a vulnerabilidade interfere nos mecanismos de gestão das unidades de saúde.

Principalmente em cenários de crise, a intervenção do governo deve se concentrar em mitigar, e não provocar, contração econômica. Mais especificamente, uma boa política de saúde – ou mudança na política de saúde existente – deve manter o fluxo de recursos necessários para sustentar os serviços aderentes à flutuação de demanda, como foi feito até o último quadrimestre de 2022, quando o Programa Previne Brasil, embora adotado, ainda não tornara efetivo o pagamento por desempenho^20,21^.

De forma geral, os gastos com a saúde devem adotar uma lógica contracíclica em tempos de crise, uma vez que o objetivo fundamental de uma política pública é garantir à população – especialmente aos mais pobres e vulneráveis – o acesso aos serviços essenciais, e isso só pode ser alcançado preservando o financiamento do setor saúde dos efeitos de choques.

Uma resposta resiliente dos sistemas de saúde a um choque envolve buscar estratégias que assegurem o desempenho das funções dos sistemas de saúde de forma sustentável, o que implica proteger o funcionamento geral do sistema de restrições orçamentárias. Além disso, a mudança de políticas de repasse de recursos não deve comprometer o provimento e a operação planejada dos serviços, nem sua capacidade de receber ajustes conforme a realidade dos territórios, sob o risco de comprometer também o desempenho geral do sistema e, por extensão, sua resiliência^22^.

O programa Previne Brasil tem sido objeto de um exaustivo debate, apontando seu caráter privatizante, seletivo e focalizado, no sentido oposto das propostas iniciais da Estratégia de Saúde da Família presentes nas Políticas Nacionais de Atenção Básica de 2006^23^ e 2012^24^, que caminhavam na direção da cobertura e acesso universais, em consonância, inclusive, com a agenda global defendida por organizações como a Organização Mundial de Saúde e a Organização das Nações Unidas^20,25^.

Os achados deste estudo evidenciam que, no que se refere à remuneração por desempenho, os territórios com as características da AP 3.1 do município do Rio de Janeiro perderiam, em valores estimados, mais de R$ 430.000,00 em função do sub-registro. Os valores encontrados mostram perdas significativas que equivalem à cobertura de financiamento de 133 equipes de saúde da família de 40 horas, 177 equipes de saúde da família de 30 horas ou 266 equipes de saúde da família de 20 horas, valores que, em um contexto histórico de subfinanciamento, não devem ser desprezados.

Chioro et al.^26^ refletem sobre as modificações das políticas públicas em função do perfil ideológico dos governos. A resiliência e a sustentabilidade do SUS dependem fundamentalmente da democracia e do apoio da população ao sistema que, para isso, precisa se mostrar cada vez mais efetivo, mesmo lidando com recursos limitados. Por outro lado, como destacado pela Organização Mundial de Saúde^27^, a introdução de reformas na forma de pagamento de serviços pode produzir consequências inesperadas e negativas. Portanto, recomenda-se que as mudanças sejam incrementais e evitem rupturas bruscas que possam causar efeitos colaterais ao sistema de saúde.

Nesse sentido, embora seja esperado que o programa Previne Brasil possa estimular aprimoramentos nos processos de registro e que a ponderação valorize regiões mais vulneráveis, o presente estudo indica que o financiamento será impactado pelo estado atual de efetividade do cadastro e deve variar substancialmente no país^28,29^.

## CONCLUSÃO

A principal contribuição deste artigo reside na apresentação de uma evidência empírica dos potenciais efeitos do sub-registro sobre o financiamento da APS. Além disso, há dois outros achados significativos – primeiro, evidencia fragilidades no processo de registro das informações de saúde inerentes a regiões vulneráveis; segundo, indica um círculo vicioso potencialmente alimentado pela alteração brusca dos condicionantes do financiamento da APS, além de potenciais consequências para os demais níveis de atenção.

Com relação às diversas dimensões que afetam o processo de registro, o presente estudo é limitado às informações disponíveis no barramento de dados do Datasus, que se refere essencialmente à capacidade institucional e pouco revela sobre o comportamento dos serviços. Nesse sentido, estudos futuros em profundidade serão pertinentes para evidenciar de maneira concreta as causas das perturbações no trabalho de coleta e análise de informações de saúde nos territórios.

É importante salientar que novas pesquisas devem ser desenvolvidas sobre os impactos financeiros que a nova forma de financiamento da atenção primária pode ter, principalmente no que tange uma maior escala, ou seja, incluindo mais municípios, assim como os demais procedimentos e indicadores que fazem parte dos indicadores do Programa Previne Brasil.
